# Docosahexaenoic acid preserves visual function by maintaining correct disc morphology in retinal photoreceptor cells

**DOI:** 10.1074/jbc.M117.790568

**Published:** 2017-06-03

**Authors:** Hideo Shindou, Hideto Koso, Junko Sasaki, Hiroki Nakanishi, Hiroshi Sagara, Koh M. Nakagawa, Yoshikazu Takahashi, Daisuke Hishikawa, Yoshiko Iizuka-Hishikawa, Fuyuki Tokumasu, Hiroshi Noguchi, Sumiko Watanabe, Takehiko Sasaki, Takao Shimizu

**Affiliations:** From the a Department of Lipid Signaling, National Center for Global Health and Medicine, Shinjuku-ku, Tokyo 162-8655,; the Departments of b Lipid Science and; j Lipidomics, Graduate School of Medicine, University of Tokyo, Bunkyo-ku, Tokyo 113-0033,; the c Agency for Medical Research and Development (AMED)–Core Research for Evolution Science and Technology (CREST), Chiyoda-ku, Tokyo 100-0004,; the d Division of Molecular and Developmental Biology and; h Medical Proteomics Laboratory, Institute of Medical Science, University of Tokyo, Shirokanedai, Minato-ku, Tokyo 108-8639,; the e Department of Medical Biology, Akita University Graduate School of Medicine, Akita 010-8543,; the f Research Center for Biosignal, Akita University Graduate School of Medicine, Akita 010-8502,; g Akita Lipid Technologies, LLC, Akita 010-0825, and; the i Institute for Solid State Physics, University of Tokyo, Kashiwa, Chiba 277-8581, Japan

**Keywords:** glycerophospholipid, membrane biophysics, membrane lipid, phospholipid turnover, retinal degeneration, DHA, LPAAT3, lysophospholipid acyltransferase

## Abstract

Docosahexaenoic acid (DHA) has essential roles in photoreceptor cells in the retina and is therefore crucial to healthy vision. Although the influence of dietary DHA on visual acuity is well known and the retina has an abundance of DHA-containing phospholipids (PL-DHA), the mechanisms associated with DHA's effects on visual function are unknown. We previously identified lysophosphatidic acid acyltransferase 3 (LPAAT3) as a PL-DHA biosynthetic enzyme. Here, using comprehensive phospholipid analyses and imaging mass spectroscopy, we found that LPAAT3 is expressed in the inner segment of photoreceptor cells and that PL-DHA disappears from the outer segment in the LPAAT3-knock-out mice. Dynamic light-scattering analysis of liposomes and molecular dynamics simulations revealed that the physical characteristics of DHA reduced membrane-bending rigidity. Following loss of PL-DHA, LPAAT3-knock-out mice exhibited abnormalities in the retinal layers, such as incomplete elongation of the outer segment and decreased thickness of the outer nuclear layers and impaired visual function, as well as disordered disc morphology in photoreceptor cells. Our results indicate that PL-DHA contributes to visual function by maintaining the disc shape in photoreceptor cells and that this is a function of DHA in the retina. This study thus provides the reason why DHA is required for visual acuity and may help inform approaches for overcoming retinal disorders associated with DHA deficiency or dysfunction.

## Introduction

Docosahexaenoic acid (DHA)^2^ plays essential roles in photoreceptor cells in acquisition of visual function. Dietary DHA modulates the maturation and survival of photoreceptor cells (1–3), and animals grown with polyunsaturated fatty acid-free diets develop abnormal electroretinograms (ERG) with decreased retinal DHA contents (4) suggesting that dietary DHA is essential for visual function. DHA is a dominant fatty acid of retinal phospholipids and affects rhodopsin content at discs, as well as photoresponses (2, 5). Many studies report the beneficial effects of DHA on visual functions (5–10); however, the molecular mechanisms and direct roles of DHA in the retina remain unknown.

All-*trans*- and 11-*cis*-retinal are flipped from the lumen to the cytoplasmic leaflet of the disc membrane via ATP-binding cassette transporter ABCA4, which is associated with Stargardt macular degeneration (11). Although essential for vision, the clearance of 11-*cis*- and all-*trans*-retinal from the photoreceptor disc membrane is important due to their highly reactive aldehyde groups. 11-*cis*-Retinal with phosphatidylethanolamine (PE) is isomerized to the all-*trans* form, which is then reduced by retinol dehydrogenase 8 for entry into the visual cycle of retinal pigment epithelial (RPE) cells (8, 12). During this step, PE-containing DHA enhances isomerization (8); however, the mechanism of how DHA affects this reaction, such as a possible binding site for DHA, is unknown. Although *in vitro* analyses using rhodopsin reconstituted into liposomes revealed that replacing C16:0-C18:1 with C18:1-C22:6 (DHA) phospholipids increased rhodopsin activation (5, 13, 14), the reason DHA has this effect remains unclear. Currently, the elucidation of molecular mechanisms associated with DHA is needed for understanding of visual functions.

Recently, we partially identified the molecular mechanism associated with DHA incorporation into phospholipids (15). DHA is first activated to DHA-CoA, which is esterified to lysophosphatidic acid to form phosphatidic acid (PA) by lysophosphatidic acid acyltransferase 3 (LPAAT3) (16, 17). The DHA-containing PA is converted into other phospholipids, such as phosphatidylcholine (PC) and PE (18). LPAAT3 is expressed in DHA-rich tissues, such as the testis (16, 19).

Here, we investigated the relationship between LPAAT3 and retinas, which contain high amounts of DHA-containing phospholipids (PL-DHA). We observed that LPAAT3 was highly expressed in the retina and that LPAAT3 deficiency dramatically lowered PL-DHA levels in the outer segment (OS) of photoreceptors and impaired visual functions. Additionally, disc morphology and/or organization in the OS of LPAAT3-knock-out (LPAAT3-KO) mice was disrupted. Cellular membrane with PL-DHA was more flexible than those with phospholipid-containing arachidonic acid (PL-AA) and other fatty acids according to analyses using dynamic light scattering (DLS) and molecular dynamics (MD) simulations. Our findings demonstrate DHA involvement in maintaining disc properties in the retina and provide insight into the roles of DHA in visual function. In the accompanying paper (56), we reported that DHA is critical also for sperm maturation.

## Results

### Lack of PL-DHA in LPAAT3-KO retinas

**Figure 1.**
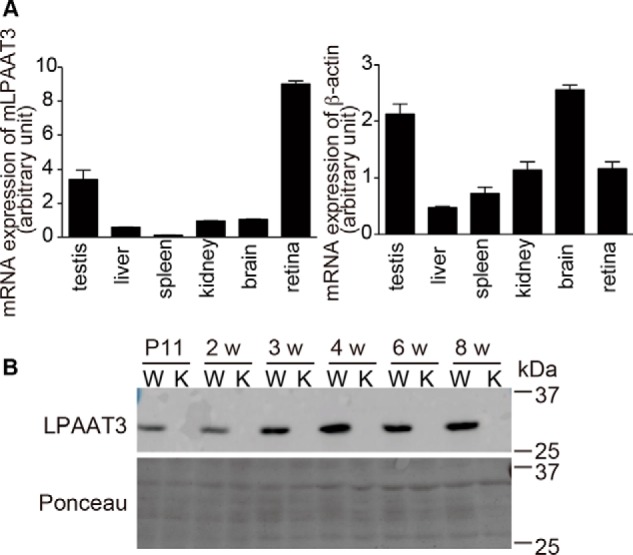
**High expression of LPAAT3 in the retina.** *A,* LPAAT3 mRNA levels in the tissues from 8-week-old mice were measured by quantitative-PCR (*left*). β-Actin level was used as a control (*right*). LPAAT3 was highly expressed in the retinas. Results are expressed as the mean ± S.E. of three independent experiments. *B,* LPAAT3 protein levels were detected by Western blot analysis using an anti-LPAAT3 antibody. LPAAT3 levels increased on an age-dependent basis. LPAAT3 was indeed disrupted in LPAAT3-KO retina. Three independent experiments were performed with similar results. *W*, WT; *K,* LPAAT3-KO.

**Figure 2.**
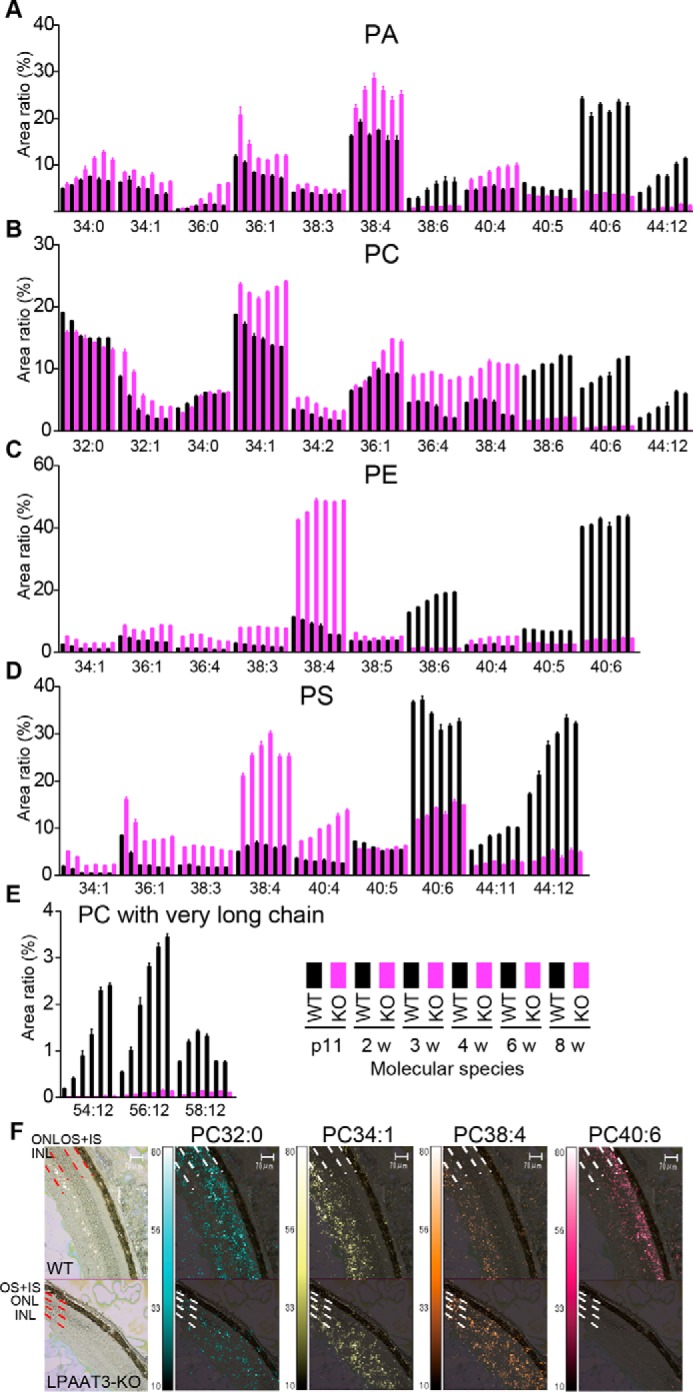
**Attenuation of PL-DHA in LPAAT3-KO retinas.** PA (*A*), PC (*B*), PE (*C*), PS (*D*), and PC with very long chain fatty acids (*E*) were measured by comprehensive phospholipid analysis and indicated by the area ratio (*y* axis, 100% indicates sum of the detected signals for each phospholipid). The *x* axis indicates the summation of fatty acid information at the *sn*-1 and *sn*-2 positions (number of carbon and double bonds, *i.e.* 34:0) in the phospholipids. 38:6, 40:6, and 44:12 in all phospholipids and very long chain in PC may contain DHA, which were suppressed in LPAAT3-KO mice. By contrast, 36:4, 38:4, and 40:4 may contain AA and were increased in LPAAT3-KO mice. 34:1 and 36:1 thought to have oleic acid were increased. These data were obtained from p11 to 8-week-old mice; WT (*black*) and LPAAT3-KO (*magenta*). Results are expressed as the mean ± S.E. of four independent experiments. *F,* PC localization of 8-week-old mice (WT, *upper*; KO, *lower*) was observed by imaging mass microscope. PC32:0, PC34:1, PC38:4, and PC40:6 are supposed to have C16:0 (palmitic acid), C18:1 (oleic acid), C20:4 (AA), and C22:6 (DHA), respectively. The signals of PC40:6 were disappeared in the OS of LPAAT3-KO retina. Imaging data of phospholipids were merged with captured light images. *Scale bar,* 70 μm. Two independent experiments were performed with similar results.

We have previously reported the age-dependent up-regulation of mouse LPAAT3 in the testis (16, 19). In this study, LPAAT3 levels were mainly evaluated in DHA-abundant mouse tissues. Quantitative-PCR analysis showed that LPAAT3 mRNA levels were higher in the retina, followed by the testis, in 8-week-old mice (Fig. 1*A*). LPAAT3 protein levels also increased in an age-dependent manner in the retina (wild-type (WT) in Fig. 1*B*). To examine the role of LPAAT3 in the retina, we constructed LPAAT3-KO mice as described in detail in the accompanying paper (56) and confirmed the absence of LPAAT3 protein in the retina (Fig. 1*B*). Comprehensive phospholipid analysis of the retina was performed using liquid chromatography-mass spectrometry (LC-MS) to detect PA, PC, PE, phosphatidylserine (PS) (Fig. 2, *A–D*), and PC with long chain fatty acids (total carbon number >54) (Fig. 2*E*), as well as phosphatidylglycerol (PG), phosphatidylinositol (PI), oxidized PC, LPA, lysophosphatidylcholine (LPC), lyso-PE, lyso-PG, lyso-PS, and lyso-PI (supplemental Fig. S1, *A–I*). In the retina of LPAAT3-KO mice, PL-DHA levels were almost abolished, whereas the PL-AA levels increased compared with WT mice. Oxidized PC generated from PC-DHA also decreased in LPAAT3-KO retinas (supplemental Fig. S1*C*). We also performed MS/MS analyses to investigate the acyl-chain composition of the representative PC species (PC38:4, PC38:6, and PC40:6). Each PC subspecies had an almost single acyl-chain composition (supplemental Fig. S1*J*). From the results, PC38:4, PC38:6, and PC40:6 were identified as PC18:0/20:4, PC16:0/22:6, and PC18:0/22:6 molecular species, respectively.

To identify the cell type in which PL-DHA was depleted in LPAAT3-KO retinas, we next examined PL-DHA localization in the retina by using an imaging mass microscope, iMscope *TRIO* (Shimadzu Corp., Kyoto, Japan). In addition to PC containing DHA and AA, representative PCs are shown in Fig. 2*F*. PC32:0, PC34:1, PC38:4, and PC40:6 are supposed to have C16:0 (palmitic acid), C18:1 (oleic acid), C20:4 (arachidonic acid, AA), and C22:6 (DHA), respectively. PC40:6 (supposed to have DHA, PC-DHA) was detected in the OS of WT retinas but disappeared in the OS of LPAAT3-KO retinas (Fig. 2*F*). By contrast, PC38:4 (PC-AA) levels increased in LPAAT3-KO retinas (Fig. 2*F*). These results were consistent with more quantitative analyses by LC-MS data (Fig. 2*B*). The levels of PC32:0 and PC34:1 were similar between WT and LPAAT3-KO retinas (Fig. 2*F*). From these results, abundant PL-DHA was located at the OS of the retina, but it almost disappeared during LPAAT3 deficiency.

### Different physical property of PL-DHA from those of PL-others (oleic acid or arachidonic acid)

**Figure 3.**
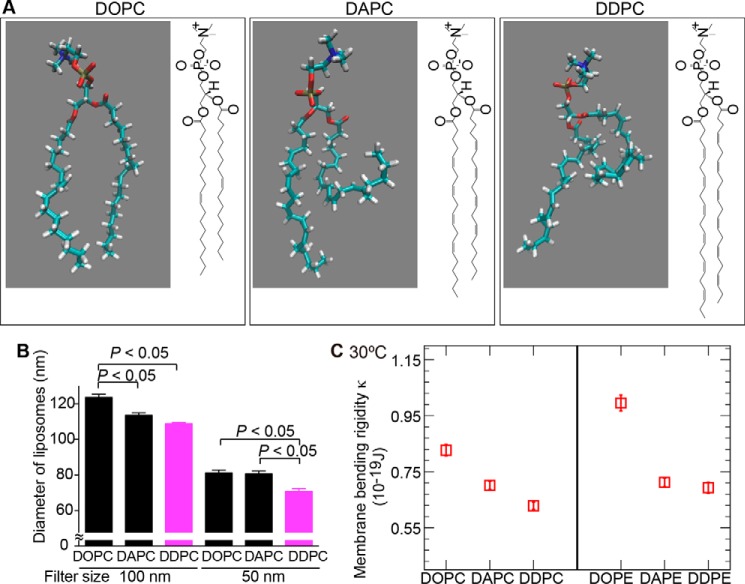
**Alternation of phospholipid physical properties by fatty acids.** *A,* single-molecule illustrations and structures of DDPC, DAPC, and DOPC. *B,* liposome sizes were measured by DLS analysis. The sizes of DDPC liposomes were smaller than those of DOPC and DAPC following passage through a 50 nm pore size filter. Results are expressed as the mean ± S.E. of three independent experiments. *p* < 0.05; one-way ANOVA, Tukey's multiple comparison test. *C,* κ was calculated by MD simulations. The κ value for DDPC was lower than that for DOPC and DAPC. DDPE also showed similar results. Statistical errors are estimated using the block average method (53).

Our observations of disrupted PL-DHA levels in LPAAT3-KO mice encouraged us to examine the physical properties of lipid bilayers composed of several PCs, including PC-DHA. Thus, we studied the size of liposomes with known phospholipid contents. Liposomes composed of di-oleoyl-PC (DOPC), di-arachidonoyl-PC (DAPC), or di-DHA-PC (DDPC) (Fig. 3*A*) were constructed and analyzed by DLS using a Zetasizer NanoZSP (Malvern Instruments Ltd., UK). Although each liposome size was adjusted by passage through a 100-nm filter, the *z*-average diameters (light-intensity weighted hydrodynamic diameters) of DAPC or DDPC liposomes were smaller than that of DOPC liposomes (Fig. 3*B*). After additional passage through a 50-nm filter, DDPC liposome size decreased significantly relative to those of DAPC and DOPC (Fig. 3*B*). These results indicate that differences in fatty acid physical characteristics directly affected each liposome size, *i.e.* the physical property of membranes containing DDPC differed from those containing DAPC or DOPC. Liposomes consisting of PC with mixed acyl chains having stearic acid at the *sn*-1 position and oleic acid, AA, or DHA at the *sn*-2 position did not show any differences (data not shown); the differences in diameter, if they existed, might have been less than the sensitivity limit of the assay.

We then calculated membrane elasticity by MD simulations using GROMACS 5.1 simulation packages (20, 21). Each membrane area expansion modulus (*K_A_*) was estimated (supplemental Fig. S2) to calculate the membrane bending rigidity (κ) for three different membranes composed of either DOPC, DAPC, or DDPC. The membrane rigidity of DDPC was lower than the rigidity of DOPC and DAPC at 30 °C, indicating that DDPC is more flexible than the others (Fig. 3*C*). These differences in κ might be attributed to the number of double bonds in the three phospholipid species. PE liposomes using di-oleoyl-PE (DOPE), di-arachidonoyl-PE (DAPE), or di-DHA-PC (DDPE) showed a similar trend (Fig. 3*C*). Although the effect of double bonds on bending rigidity was decreased at 50 and 70 °C (supplemental Fig. S2), DDPC showed the lowest κ at all three temperatures.

### Abnormality of retinal layers in LPAAT3-KO mice

**Figure 4.**
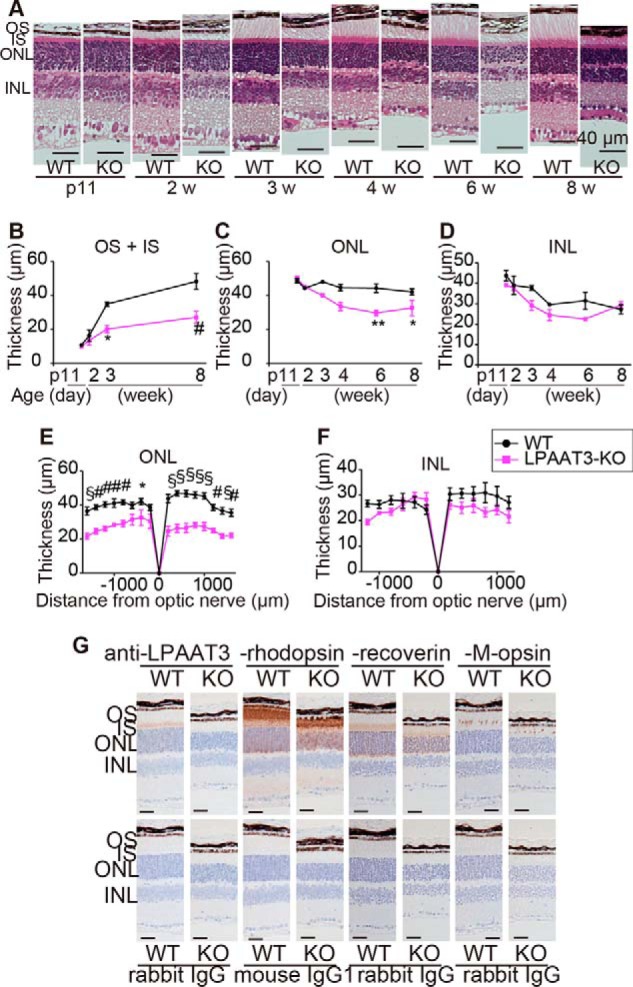
**Loss of retinal layers of LPAAT3-KO mice.** Retinal structure (*A*) and their thicknesses (*B–F*) are shown. OS + IS and ONL were thinner in LPAAT3-KO retina. Three independent experiments were performed with similar results (*A*). Results are expressed as the mean ± S.E. of three (p11 to 6 weeks (*w*)) or five (8 weeks) independent experiments (*B–F*). *, *p* < 0.05; **, *p* < 0.01; #, *p* < 0.001; §, *p* < 0.0001; two-way ANOVA, Bonferroni test. *G,* immunohistochemistry was performed to detect LPAAT3, rhodopsin, recoverin, and M-opsin in 6-week-old mice. Diaminobenzidine (*brown*) signals indicate each protein. *Scale bar,* 100 μm. Independent experiments from three mice were performed with similar results.

LPAAT3-KO retinas indicated decreases and increases of PL-DHA and PL-AA levels, respectively, with each exhibiting distinct physical properties. To examine whether the elimination of PL-DHA would affect retinal structure, we next performed histological analyses of LPAAT3-KO retinas. The thicknesses of the outer nuclear layer (ONL), inner nuclear layer (INL), inner segment (IS), and OS were comparable between WT and LPAAT3-KO retinas at ∼2 weeks following birth (Fig. 4, *A–D*), indicating that early retinal development was not significantly affected by PL-DHA deficiency. However, 3–8-week-old mice exhibited fully elongated OS with IS in WT retinas but not in LPAAT3-KO retinas (Fig. 4, *A* and *B*). This developmental benchmark in OS coincided with increased LPAAT3 expression in the retinas (Fig. 1*B*). Furthermore, the ONL in 6- and 8-week-old LPAAT3-KO retinas were thinner than those in WT retinas (Fig. 4, *A*, *C,* and *E*), whereas the INL thickness was similar between WT and LPAAT3-KO retinas (Fig. 4, *A*, *D,* and *F*).

Next, the expression patterns of LPAAT3 and markers for rod photoreceptor cells (rhodopsin and recoverin) and cone photoreceptor cells (M-opsin) were detected by immunohistochemistry. We observed that LPAAT3 was localized in the IS indicating that PL-DHA was biosynthesized by LPAAT3 in the endoplasmic reticulum and/or other organelles in photoreceptor cells (Fig. 4*G*). Rhodopsin was primarily localized to the OS of rod photoreceptor cells in WT and LPAAT3-KO retinas; however, in LPAAT3-KO retinas, rhodopsin expression may have shown a slightly disturbed pattern in ONL. By contrast, recoverin was mainly expressed in the IS of WT retinas but appeared diffusely in both the IS and OS of LPAAT3-KO retinas (Fig. 4*G*). Furthermore, M-opsin was aligned in the OS of WT retinas but exhibited fragmented signals in LPAAT3-KO retinas (Fig. 4*G*). Thus, the structures of the IS and OS were disorganized, and rhodopsin was partially stacked at ONL in LPAAT3-KO retinas.

### No influence of phototoxicity on retinal disruption in the LPAAT3-KO mice

**Figure 5.**
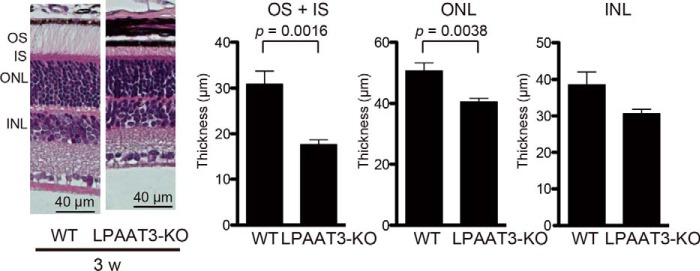
**No influence of phototoxicity on retinal disruption in the LPAAT3-KO mice.** Mice at 1–3 weeks old were maintained under dark conditions for 2 weeks, and their retinal layers were analyzed. OS/IS and ONL from LPAAT3-KO retinas were lower than those of WT retinas, with similar results to those under normal light/dark cycle conditions. LPAAT3-KO mice were not influenced by phototoxicity under the conditions. Four independent experiments were performed with similar results (hematoxylin and eosin staining). Results are expressed as the mean ± S.E. *p* value, Mann-Whitney *U* test. Experimental numbers of OS/IS were *n* = 5 in WT and *n* = 8 in LPAAT3-KO retinas from each four mice. ONL and INL were *n* = 8 from four retinas from each mouse.

Next, we examined whether the damage of retinal structure was affected by phototoxicity. ABCA4 clears 11-*cis*- and all*-trans*-retinal from the photoreceptor disc membrane to protect against phototoxicity (11). During this step, 11-*cis*-retinal combined with PE-DHA is effectively isomerized to an all-*trans* isomer by retinol dehydrogenase 8 and enters the visual cycle (8). We also investigated the effect of phototoxicity on retinal degeneration in LPAAT3-KO mice. Mice were housed under dark conditions for a 2-week period when they were 1–3 weeks old, and then their retinal layers were analyzed. The OS/IS and ONL of LPAAT3-KO retinas were lower than those of WT mice (Fig. 5) with similar results obtained under normal light/dark cycle conditions (Fig. 4, *A–F*). These results demonstrate that retinal degeneration of LPAAT3-KO mice was not caused by phototoxicity.

### Loss of visual function in LPAAT3-KO mice

**Figure 6.**
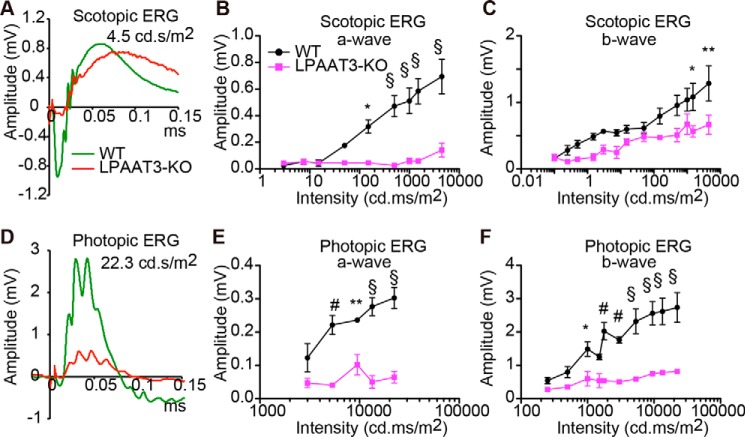
**Disorder of visual function of LPAAT3-KO mice.** ERG amplitudes at the indicated light intensities are shown at scotopic (*A–C*) and photopic (*D–F*) responses. *A* and *D,* independent experiments from three 8-week-old mice were performed with similar results. *B, C, E,* and *F,* results are expressed as the mean ± S.E. of three independent experiments. *, *p* < 0.05; **, *p* < 0.01; #, *p* < 0.001; §, *p* < 0.0001; two-way ANOVA, Bonferroni test.

To examine the effect of PL-DHA attenuation and OS damage on visual function, 8-week-old LPAAT3-KO mice were assessed by ERGs. A dark-adapted ERG revealed 80 and 50% decreases in the a- and b-wave amplitudes, respectively, in LPAAT3-KO retinas at light intensities of 4.5 cd·s/m^2^ (Fig. 6, *A–C*). Similarly, light-adapted ERG showed 80 and 70% decreases in the a- and b-waves, respectively, in LPAAT3-KO retinas at light intensities of 22.3 cd·s/m^2^ (Fig. 6, *D–F*). Therefore, these results indicate that loss of PL-DHA as a consequence of LPAAT3 deficiency resulted in visual dysfunction.

### Disruption of disc structures by PL-DHA attenuation in LPAAT3-KO retinas

**Figure 7.**
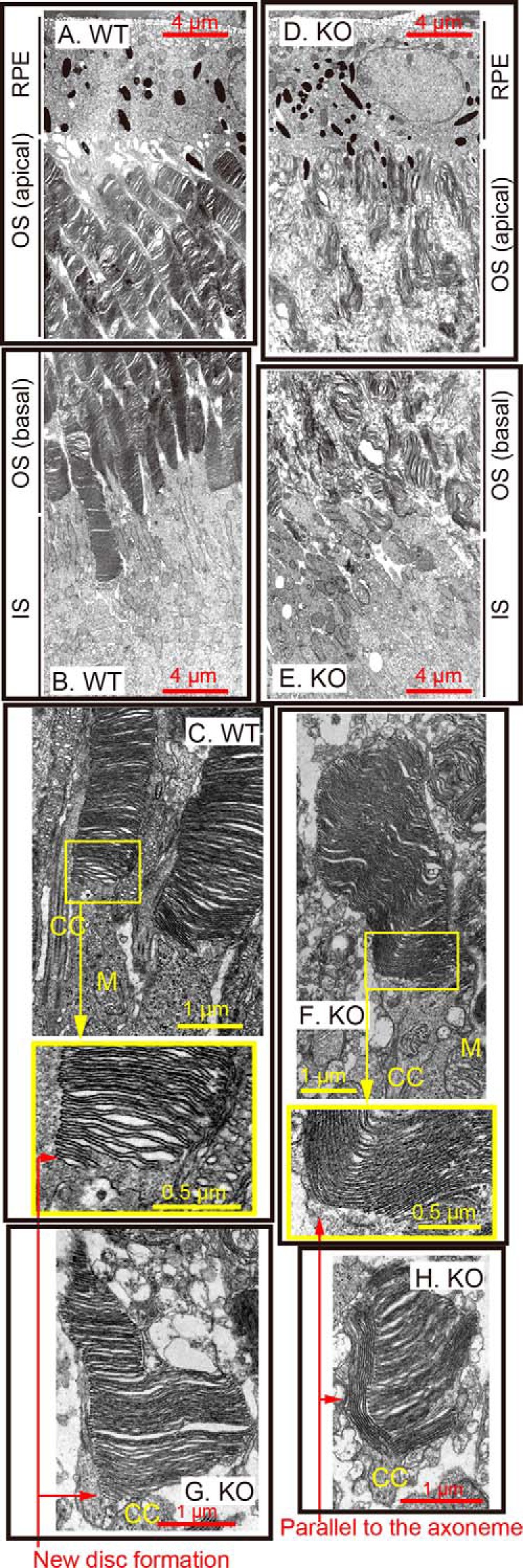
**Disc disruption in LPAAT3-KO.** Retinal structures of WT (*A–C*) and LPAAT3-KO (*D–H*) mice were observed by TEM. Abnormal disc morphogenesis was observed in LPAAT3-KO retinas. Independent experiments from three mice were performed with similar results. *CC,* connecting cilium; *M,* mitochondria.

**Figure 8.**
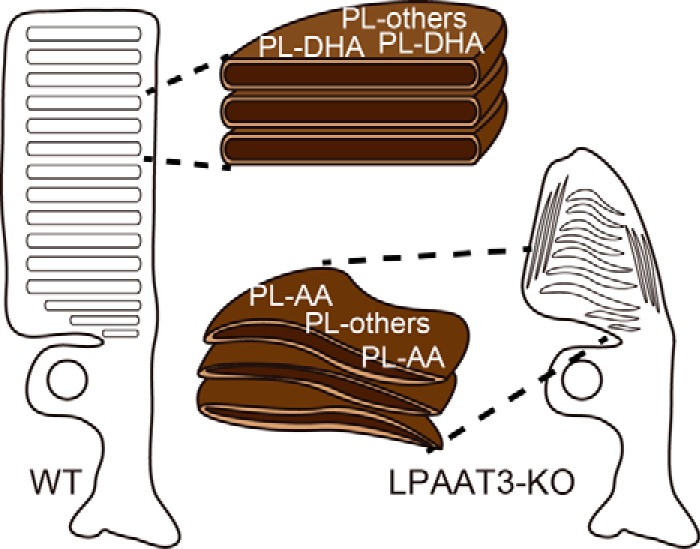
**Model figures of DHA roles.** Model figures of proposed DHA roles in photoreceptor cells. Low levels of PL-DHA in LPAAT3-KO-induced abnormal disc morphology/organization, which was not rescued by increased PL-AA levels.

We further examined the precise structure of the IS and OS of photoreceptor cells in LPAAT3-KO retinas by transmission electron microscopy (TEM). The retinas of 3-week-old mice were investigated, because the OS length was shortened significantly in LPAAT3-KO retinas according to the retinal sections in this stage (Fig. 4, *A* and *B*). At low magnification, the OS was well organized, and the disc exhibited normal formation in WT retinas (Fig. 7, *A–C*) but not in LPAAT3-KO retinas (Fig. 7, *D–H*). Highly disorganized but membranous disc-like structures were detected at the basal side of the OS in LPAAT3-KO retinas (Fig. 7*E*). However, RPE cells exhibiting normal structures may not affect OS abnormality in LPAAT3-KO retinas (Fig. 7*D*). Close examination of the IS-OS junction in LPAAT3-KO retinas revealed no drastic morphological changes in the IS and the connecting cilium (Fig. 7, *F–H*). Given that discs showed abnormal morphology compared with WT (Fig. 7*C*), but were at least formed and located at the OS in LPAAT3-KO retinas, membrane evagination and/or fusion at the base of the OS (22) might still be processed normally (*WT,* Fig. 7*C*; *KO*, Fig. 7, *F* and *G*). However, abnormal discs parallel to the axoneme, which is a hallmark of abnormal OS morphogenesis (23), were also observed in LPAAT3 KO retinas (Fig. 7, *F* and *H*). These results indicate that PL-DHA might be more essential for disc organization and/or maintenance of morphology than for new disc formation at the basal OS. Combined with DLS and MD simulation data, our results reveal that the different physical properties of phospholipids induced the distinct disc characteristics between WT and LPAAT3-KO retinas. Based on their structural flexibilities, PL-DHA but not PL-AA may be essential for disc organization/morphology (Fig. 8).

## Discussion

This study provides a molecular mechanism associated with how DHA promotes visual functions. Attenuation of PL-DHA in retina as a consequence of LPAAT3 deficiency induced visual dysfunction due to the disruption of disc morphology in photoreceptor cells. DLS analysis revealed distinct physical properties of DDPC liposomes as compared with those of DAPC liposomes according to the increased number of double bonds in fatty acids. These results from DLS analysis were supported by MD simulations. This study proposes a molecular mechanism of DHA functions in photoreceptor cells formation (Fig. 8).

In LPAAT3-KO retinas, although PL-DHA levels were strongly suppressed, PL-AA levels were increased (Fig. 2 and supplemental Fig. S1). We do not have a clear answer regarding the mechanism by which PL-AA increased; therefore, fatty acid metabolism in the LPAAT3-KO retinas requires future investigation. However, increased PL-AA did not compensate for lack of PL-DHA in disc maintenance and/or organization. One possible reason might be explained from the results of DLS analysis and MD simulations (Fig. 3, *B* and *C*). These results consistently indicate that the limit sizes of liposomes were reduced coinciding decreases with phase transition temperature, which decreases with increasing double bond number in fatty acids (24). The high flexibility resulting from fatty acid desaturation in phospholipids might contribute to the maintenance of precise disc conformations (Fig. 8). Recently, polyunsaturation of fatty acids was reported to promote fluidity on both two-dimensional (*x-y* plane) and three-dimensional (*z* direction) phospholipid planes (25). Polyunsaturated fatty acids such as DHA are either extended or bent with lower energy penalties than saturated and monounsaturated fatty acids (5, 25–28), which is consistent with our results (Fig. 3*C*). These physical properties of DHA may allow PL-DHA molecules at the highly curved edges of the disc in photoreceptor cells to maintain the disc shape (14, 25). PL-DHA in the disc may also increase the stability and function of rhodopsin (5, 13, 14, 29). Because discs also contain high amount of dipalmitoyl-PC (DPPC) (9, 30), cooperation between PL-DHA and DPPC (and also rhodopsin) might be important. DPPC is mainly biosynthesized by LPC acyltransferase 1 (LPCAT1) (15, 18, 31), the loss of which by mutation induces retinal degeneration (30).

We observed disc abnormalities as a consequence of attenuated PL-DHA levels in LPAAT3-KO retinas. Discs undergo continuous renewal throughout the animal's life span, with new membranes added at the OS base (32); however, the mechanisms by which this occurs remain controversial (22, 33, 34). A prominent feature of the OS in LPAAT3-KO retinas was the abnormal discs that were parallel rather than perpendicular to the axoneme. Similar aberrant OS morphology was reported in vesicle transporter-related protein-deficient mice such as Bardet-Biedl syndrome 4-deficient and Smad anchor for receptor activation-deficient mice (34, 35). Although the molecular mechanisms associated with disc transport (34, 35) and PL-DHA biosynthesis (this study) are different, abnormal disc development appeared to result in similar phenotypes. From our results, the physical properties of PL-DHA may contribute to the organization and/or maintenance of discs with precise structure and flexibility. DLS analysis and MD simulation studies support this hypothesis; however, we cannot exclude the possibility of vesicular protein constituents being altered in the absence of PL-DHA.

DHA is also thought to have an effect on facilitation of photoresponses (5, 13, 14). The existence of PL-DHA increases the amount and the active state of rhodopsin in the disc (36). The disc of photoreceptor cells is phagocytosed by RPE cells. Under this condition and in the presence of oxidative stress from disc phospholipids, the DHA derivative neuroprotectin D1 (NPD1) (37) is released to promote photoreceptor and RPE cell survival (38). DHA released from RPE cells is incorporated in photoreceptor cells through the adiponectin receptor 1-dependent pathway. Adiponectin receptor 1 was deficient in OS-disrupted mice (9) similar to LPAAT3-KO mice. LPC containing DHA is also supplied to RPE cells via a major facilitator superfamily domain containing 2A (Mfsd2a) (10). Although PL-DHA was not completely abolished in Mfsd2a-deficient eyes, abnormal OS structure was similar to that observed in LPAAT3-KO retinas. However, visual function of Mfsd2a-deficient mice was only slightly reduced (10). According to other literature, free non-esterified DHA may be taken up by tissues (39). Combined with the roles of PL-DHA in the storage of lipid-mediator docosanoids (37), PL-DHA may exert additional beneficial roles in visual function aside from maintaining/generating the disc.

In summary, this study proposes molecular mechanisms associated with DHA in visual function. Our findings indicate that LPAAT3-KO mice lost visual function due to abnormal disc morphology, which was caused by the reduction of PL-DHA in OS of retinas. Additionally, DLS analysis and MD simulations revealed the effects of DHA flexibility on precise disc maintenance/organization. Incorporation of DHA into phospholipids and the compensation for DHA physical functions are important for maintenance of retinal function and repair of DHA-related eye disorders. This study proposes one reason why we need DHA to see well. Studying and understanding the roles of DHA in retinas pave the way to control and preserve visual function.

## Experimental procedures

### Quantitative-PCR analysis

Total RNA was prepared (RNeasy Mini Kit; Qiagen, Hilden, Germany); cDNAs were synthesized (Superscript III; Thermo Fisher Scientific, Waltham, MA), and PCR (LightCycler System; Roche Applied Science, Basel, Switzerland) was performed using FastStart DNA Master SYBR Green I (Roche Applied Science). Primers were as follows: forward primer, 5′-ACCTATACCGCCGTATCAACTGC-3′, and reverse primer, 5′-AGTCGATCTCGAAGTTGTGGTTG-3′ for mouse LPAAT3; and forward primer, 5′-GCTGTGCTATGTTGCTCTAGACTT-3′, and reverse primer, 5′-AATTGAATGTAGTTTCATGGATGC-3′ for mouse β-actin.

### Western blot analysis

Retinas were homogenized in ice-cold buffer containing 20 mm Tris (pH 7.4), 300 mm sucrose, 50 mm β-glycerophosphate, 1 mm sodium orthovanadate, and protease inhibitor mixture Complete (Roche Applied Science). Homogenates were centrifuged at 800 × *g* for 10 min, and the supernatants were next centrifuged at 100,000 × *g* for 1 h. The resultant pellets were homogenized in ice-cold buffer containing 20 mm Tris (pH 7.4), 300 mm sucrose, 50 mm β-glycerophosphate, 1 mm sodium orthovanadate, and 1 mm EDTA. The proteins were utilized for protein quantification using protein assay solution (Bio-Rad) and Western blot analysis.

Protein samples were resolved on 10% SDS-polyacrylamide gels and electrophoretically transferred to nitrocellulose membranes (GE Healthcare, Little Chalfont, UK) using a Trans-Blot transfer cell (Bio-Rad). Membranes were blocked for >16 h with 5% skim milk (BD Biosciences) in Tris-based buffer with 0.1% Tween 20 (Wako, Osaka, Japan) at 4 °C. Anti-LPAAT3 antibody (16, 19) was diluted (1:1000) in 5% skim milk/Tris-based buffer. Horseradish peroxidase-conjugated secondary antibodies (GE Healthcare) were used at a 1:2000 dilution. ECL select Western blotting detection system (GE Healthcare) was used for chemiluminescence and was detected using ImageQuant LAS500 (GE Healthcare). Ponceau S (Sigma) staining was performed to detect proteins on the membrane as a control.

### Phospholipid analyses

Comprehensive PL analysis was described previously (40, 41). Briefly, total PLs were extracted from the retina with the Bligh-Dyer method (42). An aliquot of the lower/organic phase was evaporated to dryness under N_2_, and the residue was dissolved in methanol for liquid chromatography (LC)/mass spectrometry (MS)/MS measurements of PC and PE. To analyze PA, PS, PG, and PI, another aliquot of the same lipid extract was added with an equal volume of methanol before being loaded onto a DEAE-cellulose column (Santa Cruz Biotechnology) pre-equilibrated with chloroform. After successive washes with chloroform/methanol (1:1, v/v), the acidic PLs were eluted with chloroform/methanol/HCl/water (12:12:1:1, v/v), followed by evaporation to dryness to give a residue, which was resolved in methanol. The resultant fraction was subjected to a methylation reaction with trimethylsilyl (TMS)-diazomethane (43) before LC/MS/MS analysis.

LC-electrospray ionization-MS/MS analysis was performed with an UltiMate 3000 LC system (Thermo Fisher Scientific) equipped with HTC PAL autosampler (CTC Analytics). A 10-μl aliquot of the lipid samples was injected, and the lipids were separated on Waters X-Bridge C18 column (3.5 μm, 150 × 1.0 mm inner diameter) at room temperature (25 °C) using a gradient solvent system as follows: mobile phase A (isopropyl alcohol/methanol/water (5:1:4 v/v/v) supplemented with 5 mm ammonium formate and 0.05% ammonium hydroxide) and mobile phase B (isopropyl alcohol supplemented with 5 mm ammonium formate and 0.05% ammonium hydroxide) ratios of 70:30% (0 min), 50:50% (2 min), 20:80% (13 min), 5:95% (15–30 min), 95:5% (31–35 min), and 70:30% (35–45 min). Flow rate was 20 μl/min. PLs species was measured by the selected reaction monitoring in positive-ion mode with a triple-stage quadrupole mass spectrometer (Vantage AM, Thermo Fisher Scientific). The characteristic fragments of individual PLs were detected by the product ion scan (MS/MS mode). Chromatographic peak areas were used for comparative quantitation of each molecular species (*e.g.* 38:6 and 40:6) in a given class of the phospholipids (*e.g.* PA and PC). Peak areas of individual species were normalized against a sum of the detected signals for each sample. Major phospholipid peaks (over 5% of each total area) and major lysophospholipid peaks (over 3% of each total area) are shown in Fig. 2, *A–E,* and supplemental Fig. S1. To identify fatty acids of PC, MS/MS analyses of PC38:4, PC38:6, and PC40:6 were performed. The collision energy was set to 40 eV. The scan range of the instrument was set at *m*/*z* 200–950 in negative ion mode (44).

For imaging mass microscope, frozen sections of retinas using sodium carboxymethylcellulose were analyzed using iMScope*TRIO* (Shimadzu Corp., Kyoto, Japan) (45, 46). Sections were scanned with a focused laser (diode-pumped 355-nm Nd:YAG laser) to acquire the mass spectrum of each spot with a laser shot number of 50 per pixels and a 1000 Hz frequency. The reflection mode was applied to each measurement. The mass range was set to *m/z* 550–900 with a scan pitch of 5 μm (for ×20 magnification) pixel size. Transitions were [M + H]^+^ → *m*/*z* 734.56, 760.57, 810.58, and 834.58 for PC32:0, PC34:1, PC38:4, and PC40:6, respectively. The Imaging MS Solution (Shimadzu Corp., Kyoto, Japan) (45, 46) was used to visualize the ion images.

### Histological analysis

Collected retinas were fixed with 4% paraformaldehyde (Wako) for 24 h. After fixation, tissues were embedded in paraffin, and sections were made in the Communal Laboratory, National Center for Global Health and Medicine (Tokyo, Japan) and GenoStaff (Tokyo, Japan). Immunohistochemistry was performed at GenoStaff (Tokyo, Japan). The sections were incubated with anti-LPAAT3 rabbit (16, 19), anti-recoverin rabbit (AB5585-1; Merck Millipore, Darmstadt, Germany), or anti-M-opsin rabbit (AB5405; Merck Millipore, Darmstadt, Germany) antibody, or negative control rabbit immunoglobulin fraction (Dako X0936) at 4 °C overnight. Then, they were incubated with biotin-conjugated goat anti-rabbit IgG (Dako Japan, Tokyo, Japan) for 30 min at room temperature followed by the addition of alkaline phosphatase-conjugated streptavidin (Nichirei, Tokyo, Japan) for 5 min. Peroxidase activity was visualized by diaminobenzidine. For rhodopsin detection, sections were incubated with anti-rhodopsin mouse (GTX23267; GenTex, Rancho Cucamonga, CA) or negative control mouse IgG1 (Dako X0931) at 4 °C overnight. Then they were incubated with Block B (Nichirei, Tokyo, Japan) for 10 min at room temperature followed by the addition of Simple Stain mouse MAX-PO(M) (Nichirei, Tokyo, Japan) for 10 min. Peroxidase activity was visualized by diaminobenzidine. The microscopic examinations were performed at the Institute of Medical Science, the University of Tokyo, National Center for Global Health and Medicine, and GenoStaff. Retinal thickness was measured using a Zeiss AxioImager M1 microscope (Carl Zeiss AG, Oberkochen, Germany) and EVOS FL Auto (Thermo Fisher Scientific).

### Electroretinograms

ERGs were performed as described previously (47). Briefly, scotopic ERGs were recorded with increasing intensities of light flashes in the dark-adapted state (>12 h). Three trials were averaged for single-flash responses. Photopic ERGs were recorded after light adaptation with a background illumination of 35 cd/m^2^. Thirty two trials were averaged for single-flash responses.

### Transmission electron microscopy

Eyes were enucleated, immediately cut into hemispheres in the fixative containing 2% paraformaldehyde and 2.5% glutaraldehyde, and fixed in the same fixative for 2 h. The samples were then post-fixed in the fixative containing 2% OsO_4_, dehydrated in a graded series of ethanol, and embedded in Epon 812 resin mixture (TAAB Laboratories Equipment Ltd., Berks, UK). Ultrathin sections were created using an ultramicrotome, stained with uranyl acetate and lead citrate, and examined under an electron microscope (Hitachi H-7500, Tokyo, Japan).

### Measurement of liposome size by dynamic light-scattering analysis

Each 1 μmol of DOPC, DAPC, and DDPC (Avanti Polar Lipids, Alabaster, AL) was dried using a centrifugal concentrator VC-96W (TAITECH, Saitama, Japan) and subsequently resuspended in 1 ml of 50 mm NaCl. The liposome suspension at a final lipid concentration of 1 mm was extruded sequentially through 100-nm (pore size) polycarbonate filters by a Miniextruder (Avanti Polar Lipids). The sizes of liposomes were measured by DLS using a ZetasizerNanoZSP (Malvern Instruments, Ltd., Malvern, UK). After filtration using a 100-nm filter, liposomes were extruded again through a 50-nm filter and measured by DLS.

### Molecular dynamics simulations

MD simulations were performed on the *NPT* ensemble (*T* is constant temperature, *P* is pressure, and *N* is number of atoms) using GROMACS 5.1 simulation packages (20, 21). Lipid molecules were modeled by the recent version of CHARMM all-atom force field (CHARMM36) (48–50), and water molecules were modeled by rigid TIP3P. At 1 bar, 400 lipid molecules and 20,000 water molecules were simulated for the temperature (30, 50, and 70 °C). The temperature and pressure were controlled by the Nosé-Hoover and Parrinello-Rahman method, respectively. Newton's equation of motion was integrated using the leap-frog algorithm, and the MD time step Δ*t* was 2 fs. LINCS algorithm was applied to the bonds with hydrogens to increase Δ*t*. The total simulation time ranged from 200 to 600 ns, with the first 50 ns regarded as equilibration time. Long-range electrostatic interactions were calculated via Particle Mesh Ewald method. All initial configurations and input parameters were generated using CHARMM-GUI Membrane Builder (51, 52). Statistical errors were estimated using the block average method (53).

The membrane area expansion modulus *K_A_* was calculated from the membrane area fluctuation 〈δ*A*^2^〉 = 〈*A*^2^〉 − 〈*A*〉^2^ as shown in Equation 1,

(Eq. 1) $$K_{A}=\frac{k_{B}TA_{0}}{\langle \delta A^{2}\rangle }$$

where *A*_0_ is the area of tensionless membrane.

The bending rigidity κ was estimated (54) as shown in Equation 2,

(Eq. 2) $$\kappa =\frac{1}{24}K_{A}{\left(d-d_{0}\right)}^{2}$$

where *d* − *d*^0^ is the actual bending thickness, and *d*_0_ is estimated at 1 nm (54). *d* is the bilayer thickness, which is calculated from the peak-to-peak distance of phosphorus density profile.

### Animals

LPAAT3-KO mice (56) without Rd8 mutation (55) were selected after mating with C57BL/6J mice (CLEA Japan, Inc., Tokyo, Japan), because LPAAT3-KO mice were constructed using C57BL/6N ES cells.

All animal experiments were approved by and performed in accordance with the guidelines of the Animal Research Committee of the National Center for Global Health and Medicine (12053, 13009, 14045, 15037, and 16062), and the animal experimentation committee (P11-045) and Institute of Medical Science (PA13-105), University of Tokyo. All experiments of gene recombination were approved by and performed in accordance with the guidelines of the Biosafety Committee of National Center for Global Health and Medicine (27-P-044).

### Statistical analysis

All statistical calculations were performed using Prism 5 (GraphPad Software, La Jolla, CA).

## Author contributions

H. Shindou designed the study, performed the experiments, analyzed the data, and wrote the manuscript. H. K. designed and performed the experiments. H. Sagara performed the electron microscopic experiments. K.-M. N. and H. Noguchi performed the MD simulations. H. Nakanishi performed the lipid analyses. Y. T. maintained the deficient mice, performed experiments, and analyzed data. F. T. supported the DLS analysis. J. S. generated the LPAAT3-KO mice and D. H. and Y. I.-H. maintained them. S. W. and T. Sasaki. designed the study. T. Shimizu designed and wrote the manuscript. All authors assisted in manuscript editing.

## Supplementary Material

Supplemental Data

## Footnotes

2 The abbreviations used are:

DHA: docosahexaenoic acid
ERG: electroretinogram
PE: phosphatidylethanolamine
RPE: retinal pigment epithelial
LPC: lysophosphatidylcholine
PA: phosphatidic acid
PC: phosphatidylcholine
PL-DHA: DHA-containing phospholipid
OS: outer segment
PL: phospholipid
DLS: dynamic light scattering
MD: molecular dynamics
PS: phosphatidylserine
PG: phosphatidylglycerol
PI: phosphatidylinositol
AA: arachidonic acid
DOPC: di-oleoyl-PC
DAPC: di-arachidonoyl-PC
DDPC: di-DHA-PC (DDPC)
DOPE: di-oleoyl-PE
DDPE: di-DHA-PC
ONL: outer nuclear layer
INL: inner nuclear layer
IS: inner segment
TEM: transmission electron microscopy
DPPC: dipalmitoyl-PC
ANOVA: analysis of variance
cd: candela.
