# Supplementary material for: Docosahexaenoic acid preserves visual function by maintaining correct disc morphology in retinal photoreceptor cells
Source: J Biol Chem. 2017 Jun 3;292(29):12054–64. doi: 10.1074/jbc.M117.790568 (PMC5519357; doi:10.1074/jbc.M117.790568)
Supplement: Supplemental Data [file supp_292_29_12054__index.html]

Docosahexaenoic acid preserves visual function by maintaining correct disc morphology in retinal photoreceptor cells — Docosahexaenoic acid preserves visual function by maintaining correct disc morphology in retinal photoreceptor cells — Role of LPAAT3 in the retina — Supplemental Data 

# Docosahexaenoic acid preserves visual function by maintaining correct disc morphology in retinal photoreceptor cells

## Supplemental Data

- Fig. S1 (.pdf, 258 KB) - Decrease of PL-DHA of LPAAT3-KO retinas.
- Fig. S2 (.pdf, 159 KB) - Area expansion modulus and membrane bending rigidity at 50????C and 70????C.
