# Supplementary material for: Docosahexaenoic acid preserves visual function by maintaining correct disc morphology in retinal photoreceptor cells
Source: J Biol Chem. 2017 Jun 3;292(29):12054–64. doi: 10.1074/jbc.M117.790568 (PMC5519357; doi:10.1074/jbc.M117.790568)
Supplement: Supplemental Data [file 10.1074_M117.790568_jbc.M117.790568-1.pdf]

Fig. S1

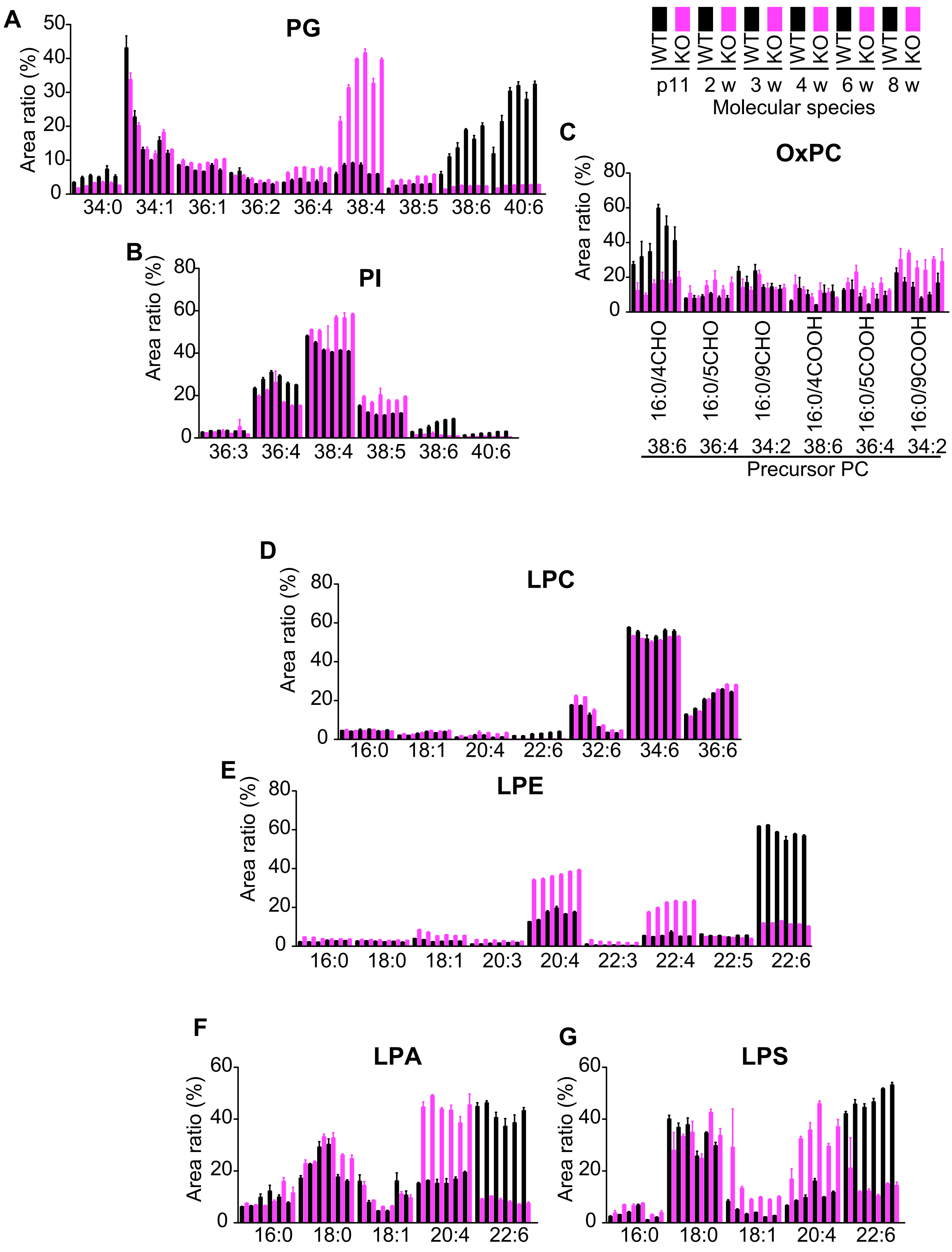
A
PG
Area ratio (%)
50
40
30
20
10
0
34:0 34:1 36:1 36:2 36:4 38:4 38:5 38:6 40:6
WT KO WT KO WT KO WT KO WT KO WT KO
p11 2 w 3 w 4 w 6 w 8 w
Molecular species
B
PI
80
60
40
20
0
36:3 36:4 38:4 38:5 38:6 40:6
C
OxPC
16:0/4CHO
16:0/5CHO
16:0/9CHO
16:0/4COOH
16:0/5COOH
16:0/9COOH
38:6 36:4 34:2 38:6 36:4 34:2
Precursor PC
D
LPC
16:0 18:1 20:4 22:6 32:6 34:6 36:6
E
LPE
16:0 18:0 18:1 20:3 20:4 22:3 22:4 22:5 22:6
F
LPA
60
16:0 18:0 18:1 20:4 22:6
G
LPS
16:0 18:0 18:1 20:4 22:6

Fig. S1H-J

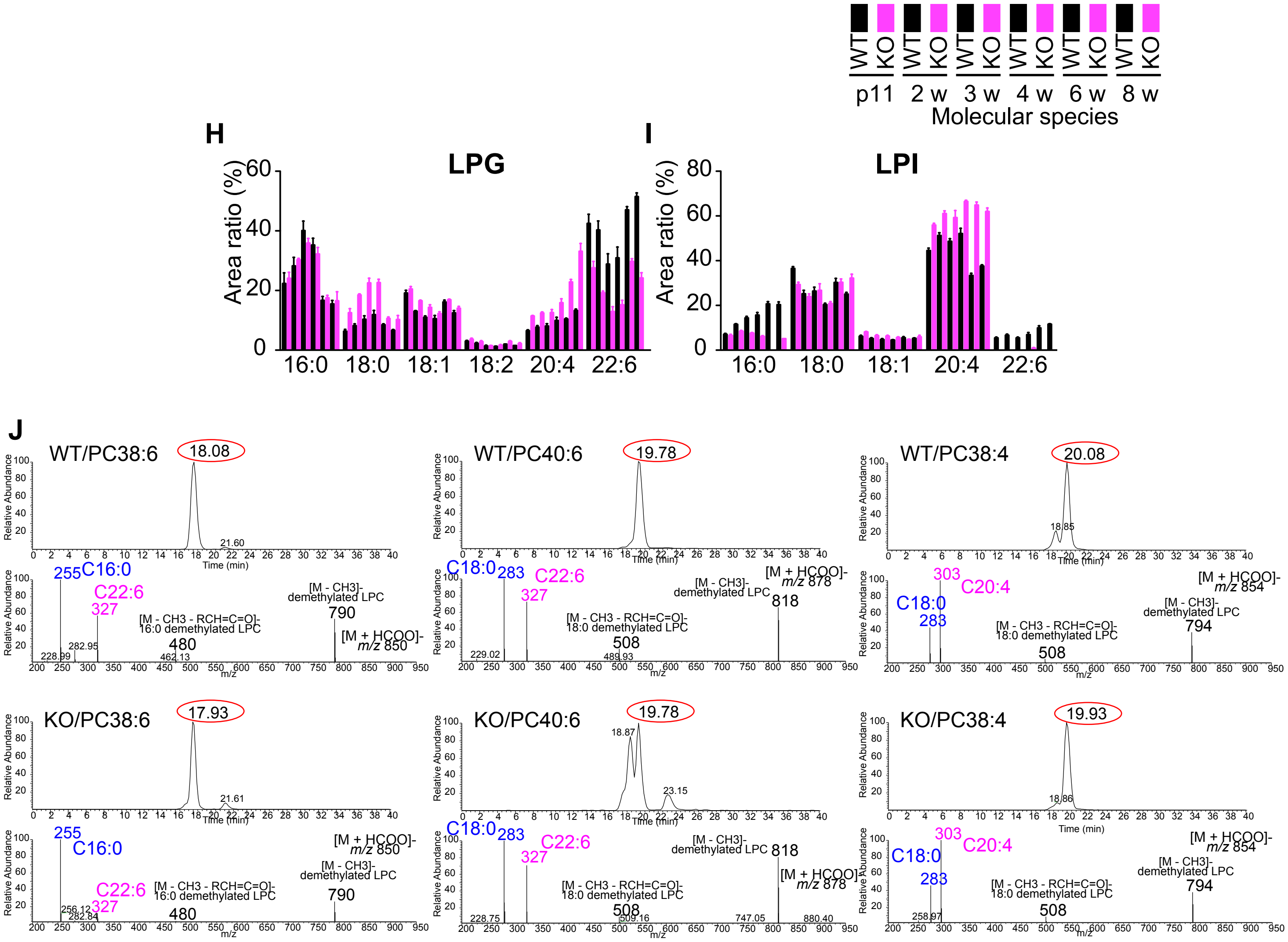

Fig. S1. Decrease of PL-DHA of LPAAT3-KO retinas.

PG (A), PI (B), oxidized PC, OxPC (C), LPC (D), LPE (E), LPA (F), LPS (G), LPG (H), and LPI (I) were measured by comprehensive phospholipid analysis and indicated by area ratio in the Y-axis (100% indicates sum of the detected signals for each phospholipid). Numbers in the X-axis indicate the summation of fatty acid information at the sn-1 and sn-2 positions (number of carbons and double bonds, i.e., 34:0) in phospholipids. Lysophospholipids (D-I) possess a single fatty acid. 38:6, 40:6, and 44:12 in all phospholipids may contain DHA, which were suppressed in LPAAT3-KO mice. By contrast, 36:4, 38:4, and 40:4 may have AA were increased in LPAAT3-KO mice. These data were obtained from p11 to 8 week-old mice; WT (black) and LPAAT3-KO (magenta). Results are expressed as the mean + SE of four independent experiments. (J) To investigate the acyl-chain composition of the representative PC species (PC38:4, PC38:6, and PC40:6, red circles), we performed MS/MS analyses of the PCs. (i) PC38:6; MS/MS of m/z 850 (PC38:6) yielded m/z 480 (acyl 16:0 demethylated LPC, loss of ketene, [M - CH3 - C18H37CH=C=O] –), m/z 255 (C16:0 FA fragment ion), and m/z 327 (C22:6 FA fragment ion). (ii)PC40:6; MS/MS of m/z 878 (PC40:6) yielded m/z 508 ([M - CH3 – C22H45CH=C=O] –), m/z 283 (C18:0 FA fragment ion), and m/z 327 (C22:6 FA fragment ion). (iii) PC38:4; MS/MS of m/z 854 (PC38:4) yielded m/z 508 ([M - CH3 – C22H45CH=C=O] –), m/z 283 (C18:0 FA fragment ion), and m/z 303 (C20:4 FA fragment ion). Thus, the position of the sn-1/2 acyl moieties of PC could be identified from these LPC-related fragment ions and fatty acid fragment ions.Each PC subspecies had an almost single acyl-chain composition, although amounts of PC38:6 and PC40:6 in LPAAT3-KO retina were low. Data were obtained from mixed samples from four
