# Supplementary material for: Docosahexaenoic acid preserves visual function by maintaining correct disc morphology in retinal photoreceptor cells
Source: J Biol Chem. 2017 Jun 3;292(29):12054–64. doi: 10.1074/jbc.M117.790568 (PMC5519357; doi:10.1074/jbc.M117.790568)
Supplement: Supplemental Data [file 10.1074_M117.790568_jbc.M117.790568-2.pdf]

**Fig. S2**

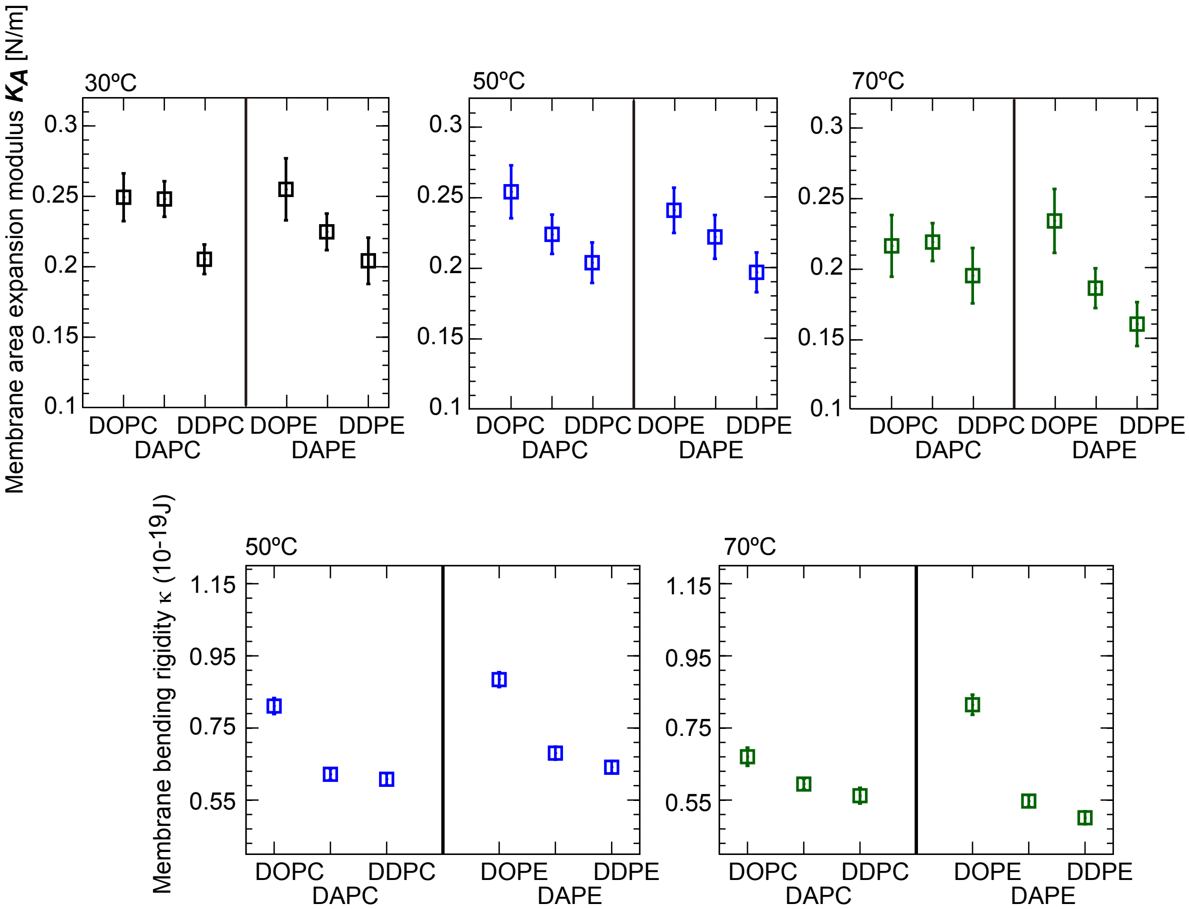


Fig. S2. Area expansion modulus and membrane bending rigidity at 50ºC and 70ºC.
The area expansion modulus KA at 30ºC, 50ºC, and 70ºC, and membrane bending rigidity κ at 50ºC and 70ºC were calculated by MD simulations. Although the differences at 50ºC and 70ºC were smaller than at 30ºC (Fig. 2C), the lowest values were observed in DDPC. Effect of double bond number in fatty acids on κ and KA value decreased at higher temperatures. DDPE also showed similar results with those of DDPC. Statistical errors are estimated using the block average method (1).


(1) Allen, M.P., ＆ Tildesley, D.J. (1989) Computer simulation of liquids. Oxford university press.
